# Examining Incentives to Promote Physical Activity Maintenance Among Hospital Employees Not Achieving 10,000 Daily Steps: A Web-Based Randomized Controlled Trial Protocol

**DOI:** 10.2196/resprot.6285

**Published:** 2016-12-12

**Authors:** Marc Mitchell, Lauren White, Paul Oh, Matthew Kwan, Peter Gove, Tricia Leahey, Guy Faulkner

**Affiliations:** ^1^ Toronto Rehabilitation Institute University Health Network Toronto, ON Canada; ^2^ Faculty of Kinesiology and Physical Education University of Toronto Toronto, ON Canada; ^3^ Department of Family Medicine McMaster University Hamilton, ON Canada; ^4^ Green Shield Canada Inc Toronto, ON Canada; ^5^ University of Connecticut Storrs, CT United States; ^6^ School of Kinesiology University of British Columbia Vancouver, BC Canada

**Keywords:** financial health incentives, physical activity, behavioral economics, randomized controlled trial, workplace health

## Abstract

**Background:**

The economic burden of physical inactivity in Canada is estimated at Can $6.8 billion (US $5 billion) per year. Employers bear a substantial proportion of the economic costs, as they pay more for inactive workers in health care and other organizational costs. In response, many Canadian employers offer wellness programs, though these are often underutilized. While financial health incentives have been proposed as one way of increasing participation, their longer term effects (ie postintervention effects) are not clear.

**Objective:**

The objective of this paper is to outline the methodology for a randomized control trial (RCT) examining the longer term impact of an existing physical activity promotion program that is enhanced by adding guaranteed rewards (Can $1 [US $0.74] per day step goal met) in a lower active hospital employee population (less than 10,000 steps per day).

**Methods:**

A 12-week, parallel-arm RCT (with a 12-week postintervention follow-up) will be employed. Employees using Change4Life (a fully automated, incentive-based wellness program) and accumulating fewer than 10,000 steps per day at baseline (weeks 1 to 2) will be randomly allocated (1:1) to standard care (wellness program, accelerometer) or an intervention group (standard care plus guaranteed incentives). All study participants will be asked to wear the accelerometer and synchronize it to Change4Life daily, although only intervention group participants will receive guaranteed incentives for reaching tailored daily step count goals (Can $1 [US $0.74] per day; weeks 3 to 12). The primary study outcome will be mean proportion of participant-days step goal reached during the postintervention follow-up period (week 24). Mean proportion of participant-days step goal reached during the intervention period (week 12) will be a secondary outcome.

**Results:**

Enrollment for the study will be completed in February 2017. Data analysis will commence in September 2017. Study results are to be published in the winter of 2018.

**Conclusions:**

This protocol was designed to examine the impact of guaranteed rewards on physical activity maintenance in lower active hospital employees.

**ClinicalTrial:**

ClinicalTrials.gov NCT02638675; https://clinicaltrials.gov/ct2/show/NCT0 2638675 (Archived by WebCite at http://www.webcitation.org/6g4pvZvhW)

## Introduction

There is a substantial economic burden associated with physical inactivity in Canada [1,2]. According to Janssen [1], the cost of inactivity is Can $6.8 billion (US $5.0 billion) per year, posing a significant threat to the sustainability of the Canadian health care system. On the other hand, the projected cost savings of increasing the proportion of Canadians who meet physical activity guidelines (ie, 150 minutes of moderate-vigorous physical activity [MVPA] per week, or roughly 10,000 steps per day) by just 1% is Can $2.1 billion (US $1.6 billion) per year [3]. Notably, significant reductions in health risk and associated costs and improvements in quality of life are seen when inactive (<5000 steps per day) and low active (5000 to 7499 steps per day) adults become a little more active (eg, 1000 more steps per day) [3,4]. The workplace is an ideal setting for physical activity promotion since Canadian jobs are increasingly desk-based and sedentary [5].

Not surprisingly, Canadian employers bear a significant proportion of the inactivity burden because they pay more for lower active (<10,000 steps per day) workers in health care expenses [6]. For instance, while Canadian provincial and territorial governments cover hospital- and physician-related medical costs, employers in Canada often subsidize other medical expenses, one of the costliest being chronic disease–related medications [6]. A study by Wang and colleagues [7] helps to illustrate the economic benefit of a more physically active employee population. This study found that moderately active (1 to 2 times per week) and very active (3 or more times per week) employees had Can $250 (US $185) less paid health care costs annually when compared to their sedentary counterparts (0 times per week). In addition, wellness initiatives that increase employees’ physical activity have been shown to reduce absenteeism, presenteeism, and turnover [8-11]. Finally, according to health care surveys by Towers Watson [12] and Sanofi [6], Canadian employers have a vested interest in employees’ physical activity levels given the positive effects on organizational costs (eg, health care expenses) and performance (eg, presenteeism).

As a result, the majority (72%) of large Canadian companies now offer wellness programs to help reduce overall health care spending and increase productivity [6]. For participating employees, such programs have been associated with a reduced risk of chronic illness and lower medical claim costs [13,14]. However, these programs are chronically underutilized. In Canada, wellness program participation rates are extremely low, less than 10% [6]. This means that more than 90% of eligible Canadian workers are not reaping the benefits of company-sponsored wellness programming. Behavioral economics, a new branch of economics that is complemented by insights from psychology, has stimulated renewed interest in financial health incentives as a means to increase wellness program participation [5,15-17].

Behavioral economics recognizes that human decisions are biased in systematic ways and that that these “decision biases” can be leveraged to facilitate healthy decision making [18]. For example, according to behavioral economics, increasing the immediately rewarding aspects of a healthy behavior (eg, with a financial incentive) may offset the so-called “present bias” where people tend to overweigh the immediate costs (and discount the future benefits) of those behaviors (eg, time out of a busy schedule to exercise) [19]. Systematic reviews by Mitchell et al [20] and Strohacker et al [21] support the theory suggesting that incentives generally stimulate physical activity in the short-term (less than 3 months) and while incentives are in place. These reviews also suggest that not enough studies have examined the longer term (ie, postintervention) impact of incentives on physical activity to draw conclusions about sustained effects [20,21]—an issue of particular interest to Canadian employers looking to deliver cost-effective incentive-based wellness programs [5,6].

Of the randomized controlled trials (RCTs) that have recently examined this issue [22-26], 4 have observed a regression to baseline behaviors after incentive removal [22-24,26] and only 1 has demonstrated persisting physical activity [25]. One reason for this may have to do with the limited application of health behavior change theories in the design of incentive programs [19]. It is increasingly suggested that for incentives to both stimulate and sustain health behavior change they should be grounded in theory [19,27,28]. In the single “positive” study, behavioral maintenance (ie, 16-week follow-up) was driven entirely by the lower active subgroup (ie, university students visiting the gym fewer than once per week at baseline)—possibly because exposure to the new behavior (ie, gym attendance) led to increased confidence to perform that behavior [25]. This presumption aligns well with self-determination theory, a global theory of motivation focused on the extent to which behaviors are done volitionally, which suggests that incentive programs designed to increase a person’s confidence are more likely to foster self-determined (or intrinsic) motivation [29,30]—a key driver of sustained physical activity [30].

Realizing the potential of incentives to promote sustained physical activity, therefore, will be contingent on research that improves the understanding of theoretical (eg, self-determined motivation) and contextual (eg, target group characteristics) factors that may influence incentive program effectiveness. The purpose of this protocol is to outline the design of an RCT that will examine the longer term effects of an existing physical activity promotion program that is enhanced by guaranteed incentives for lower active employees only in a real-world, ecological setting. We hypothesize that targeting lower active employees with incentives for tailored daily step goals (in addition to the generic, one-size-fits-all approach to goal setting that is typically used) may be more likely to create mastery experiences, increase confidence, and promote physical activity maintenance.

## Methods

### Study Design

**Figure 1:**
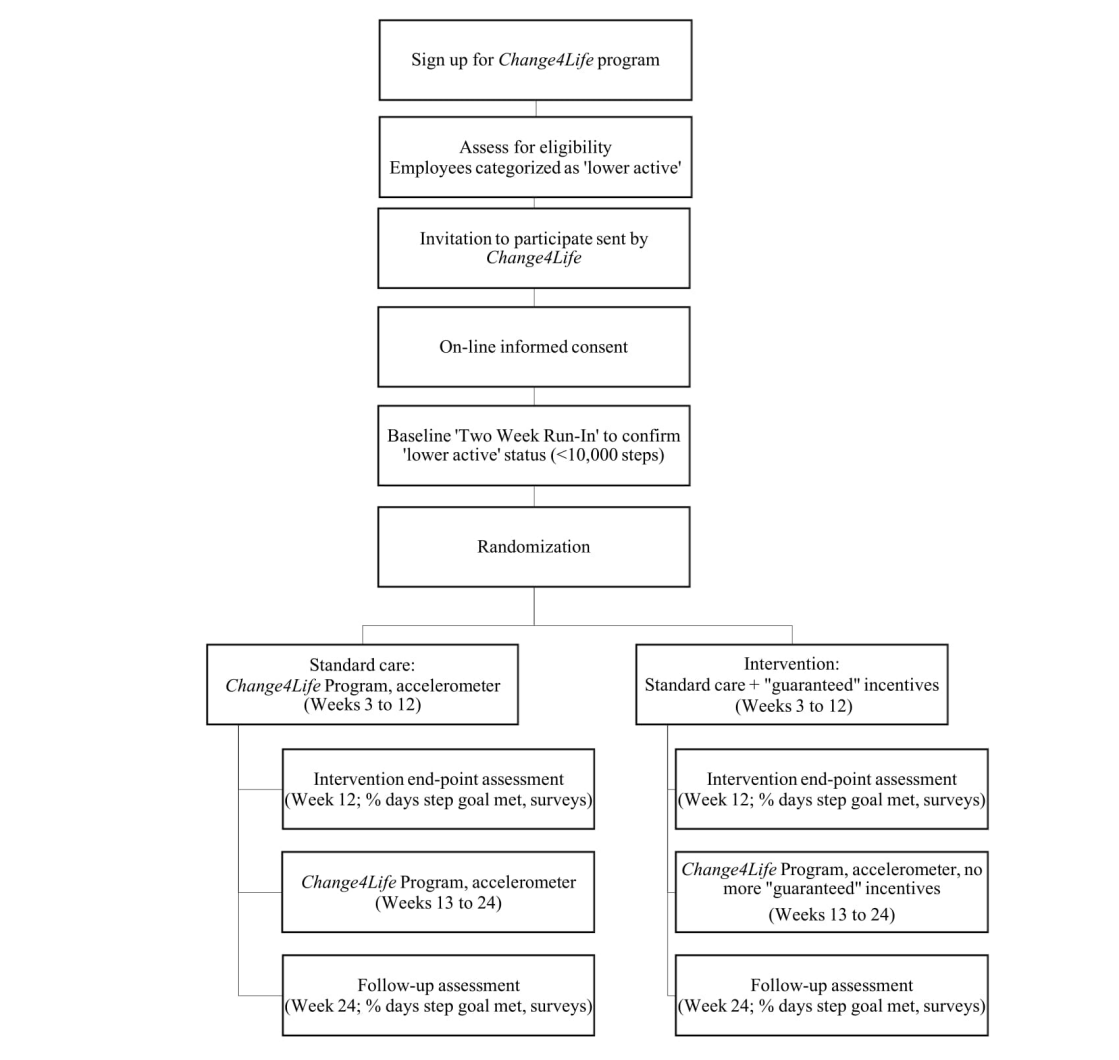
Study flowchart.

A 12-week, parallel-arm RCT with a 12-week postintervention follow-up will be employed to examine the impact of an enhanced (Can $1 [US $0.74] per day) incentive program on objectively measured physical activity among lower active employees (<10,000 steps per day) within a large Canadian hospital network. See the study flow chart in Figure 1, including an overview of the enrollment process and assessments.

### Study Population and Recruitment

This study will specifically target lower active employees (<10,000 steps per day). Hospital employees (including health care professionals and administrative and business support personnel) enrolled in the Change4Life program will be invited to participate via website notifications and hospital newsletters. Only data from participants who accumulate less than 10,000 steps per day during the “2-Week Run-In” period will be included in the analysis. Further eligibility criteria will include: 18 years of age or older, English speaking, ready Internet access, and without medical conditions exacerbated by physical activity as assessed by the Physical Activity Readiness Questionnaire Plus. Eligible participants will be asked to provide their expressed consent using the online consent form.

### Change4Life

**Figure 2:**
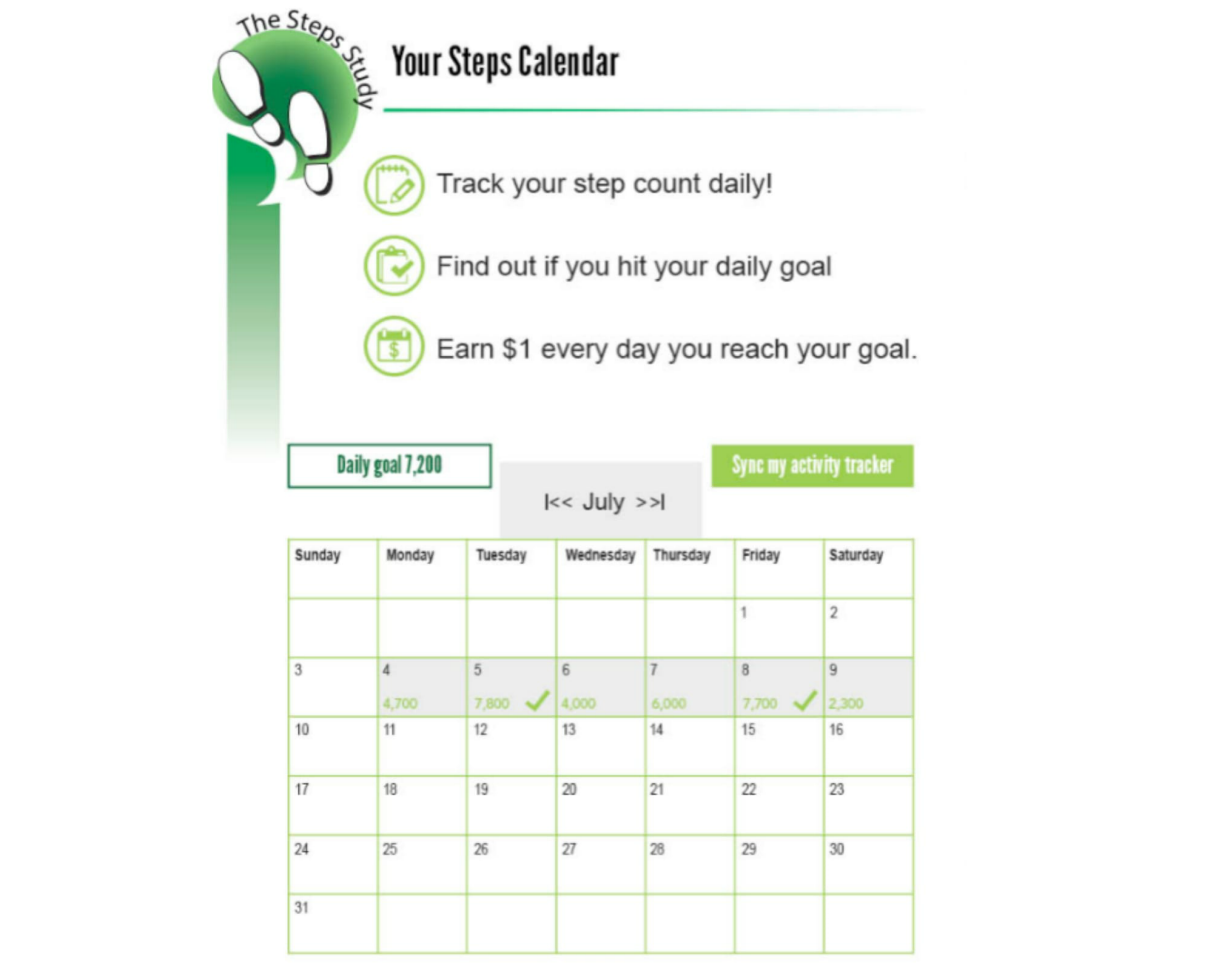
Change4Life Steps Study calendar (ie, tracking page).

Change4Life, a Web- and incentive-based health education and behavior change program, was launched in May 2015 at the hospital network. The hospital network is offering Change4Life to 6500 full-time employees across 8 worksites. Specifically, the Web-based wellness program offers educational information relating to chronic disease prevention via learning modules (ie, series of short articles and quizzes). All employees who sign up for Change4Life are rewarded with points for completing these modules as well as for setting health-related goals, self-reporting health behaviors/outcomes, identifying barriers, and creating action plans—self-regulatory behaviors that have been theorized and empirically proven to promote sustained health behavior change (see the Change4Life Steps Study calendar in Figure 2) [29,30]. Using points, employees may “purchase” ballots in the Change4Life reward store and enter into drawings for health-promoting products (eg, groceries, exercise equipment). At the hospital network, Change4Life operates as a minimal chance-based incentive program (low frequency, low magnitude rewards), where participants have less than a 1 in 100 chance of winning reward drawings that generally range in value from Can $5 to $20 (US $3.70-$14.80).

### Standard Care Group

Standard care group participants will have access to Change4Life and will receive the standard minimal chance-based incentives (ie, less than 1 in 100 chance of earning Can $5 to $20 [US $3.70-$14.80] vouchers) for completing learning modules and health tasks. In addition, standard care participants will be asked to wear the accelerometer, which tracks steps and 10-minute bouts of MVPA per day, synchronize the device to the Change4Life program daily, and reach tailored daily step count goals for 10 weeks (weeks 3 to 12). Standard care participants will be instructed to increase their daily step counts by 1000 steps above their baseline average every 2 weeks until they reach the target goal of 3000 extra steps at week 7 (see Table 1). Change4Life will automatically calculate the average baseline value in the early hours of day 1, week 3 using data uploaded the previous 2 weeks (the Run-In period)—days with step counts less than 100 or greater than 50,000 will not be included in calculations [31]. This tailored and graded approach to setting step goals is more realistic than the traditional 10,000 step target (ie, the average Canadian adults accumulates only about 5000 steps per day) [32], may increase chances of intervention success (versus offering a lofty 10,000 step per day goal for everyone), and has worked well in employee populations in the past [33].

**Table 1:** Daily step count goals across the Change4Life program.

Study week	Step count goal
Weeks 1-2	2-Week Run-In (to confirm lower active status and calculate daily step count average)
Weeks 3-4	Increase daily steps by 1000 above baseline average
Weeks 5-6	Increase daily steps by 2000 above baseline average
Weeks 7-8	Increase daily steps by 3000 above baseline average
Weeks 9-12	Maintain steps at 3000 above baseline average

### Intervention Group

The only difference between the standard care and intervention groups will be the addition of the guaranteed incentive (Can $1 [US $0.74] per day). During the intervention period (weeks 3 to 12), intervention group participants will be eligible to earn Can $1 (US $0.74) in vouchers (eg, groceries, coffee, or movies) each time tailored daily step goals are achieved. The total amount available over the intervention period will be Can $70 (US $51.82). Previous research suggests that as little as Can $6.75 (US $5) per week may be sufficient to produce favorable lifestyle health behavior changes [34], thus Can $7 (US $5.18) per week (Can $1 [US $0.74] per day) was chosen as the size of the incentive. During the study follow-up period (weeks 13 to 24), intervention participants will no longer receive the per day reward for reaching daily step goals.

### Outcome Measures

The primary study outcome will be the mean proportion of participant-days that step goals are achieved during week 24 (the postintervention follow-up assessment period, T3). Mean proportion of participant-days that step goals are achieved during week 12 (intervention end point, T2) and volume of steps and 10-minute bouts of MVPA per week at T2 and T3 will be secondary outcomes. Physical activity will be objectively assessed using the StepsCount Piezo Rx accelerometer (StepsCount Inc, Deep River, ON, Canada). The Piezo Rx is a medical-grade device with a single axis piezoelectric sensor. This device has been found to be valid in calculating step counts and MVPA among adult participants [35,36]. Standard care and intervention participants will be encouraged to wear their accelerometer and synchronize it to the Change4Life program during the baseline 2-Week Run-In period (T1), as well as at the intervention end point (T2), and follow-up assessment (T3) with Can $10 (US $7.40) study retention vouchers; these vouchers for assessment completion will help minimize dropout and will not be contingent on step goal achievement.

The adherence outcome variables will be mean number of missing step count entries per week as well as mean number of Change4Life website log-ins in general. Participants’ self-determined motivation to exercise will be examined using the Behavioral Regulation to Exercise Questionnaire-3 (BREQ-3) [37,38]. Participants’ walking self-efficacy will be assessed using a modified version of the Self-Efficacy for Exercise Scale (SEE Scale) [39]. The BREQ-3 and the SEE Scale will be administered online at baseline (T1), intervention end point (T2), and during the follow-up assessment period (T3). The differential impact of the Can $1 (US $0.74) per day incentive on the various physical activity outcomes will be explored. For instance, we may find that while the guaranteed incentives stimulate step goal achievement at T2, they also undermine self-determined motivation and thus the prospect that people will continue to exercise after the incentive is removed.

### Sample Size

Sample size calculations indicate that a final sample of 158 participants (79 per group) ensures 80% power (*P*<.05; 2-tailed) to detect a 0.20 difference in the mean proportion of participant-days step goals are reached between intervention and standard care groups for week 24 [24]. This calculation assumes that the mean proportion of participant-days step goal achieved in the intervention group in week 24 will be 0.40 (vs 0.20 in the standard care group) [24]. On the basis of data published by Patel et al [22] this difference translates into a relative effect size of 0.40. The participant enrollment target will be increased to 174 to account for a potential 10% dropout rate, a rate that has been reported by other similar studies [22,26].

### Randomization and Blinding

Employees accumulating fewer than 10,000 steps per day during the baseline period (study weeks 1 and 2) will be randomized using a single, constant allocation ratio (1:1) to standard care or intervention groups. Randomization will occur using an online random number generator [40]. Participants will not be blinded to study group allocation. The research analysts will be blinded to group allocation until after the study is completed.

### Statistical Analyses

For each participant on each day of the study (participant-day level) continuous step count data will be obtained and screened for outliers (less than 100 steps per day, more than 50,000 steps per day) [31]. If participants did not synchronize their accelerometer for at least 3 separate workdays at T2 and T3, the last observation will be carried forward using T1 or T2 means [33]. This procedure conservatively assumes no change in variables and allows analysis by intention-to-treat. The step count data will then be dichotomized at the participant-day level to create a binary variable where participants achieved (value = 1) or did not achieve (value = 0) their step goal. Using this binary variable, the mean proportion of participant-days where step goals were achieved at week 24 will be compared.

SPSS version 21.0 (IBM Corp) will be used to fit a generalized linear model with participant random effects, a random intercept, time-fixed effects (T1-T3), and treatment-fixed effects (by study group). A binomial distribution with logit link for models using the binary outcome will be used to estimate the adjusted difference in the proportion of participant-days step goal achieved, and the bootstrap procedure and resampling of participants will be used to obtain 95% confidence intervals and *P* values. Comparisons across study groups will be adjusted for mean steps per day (baseline), age, gender, and income since these have moderated incentive-effects in the past [41]. The same procedures will be used to analyze T2 (ie, intervention end) binary data.

For continuous step count (ie, mean steps per day) and bout minutes (ie, total minutes of MVPA in 10-minute bouts per week) data at T2 and T3, a generalized linear model will be used, as above, except the difference in steps per day and MVPA bout minutes between groups will be obtained using least-squares means. Also, a repeated measures analysis of variance using linear mixed models with first order autoregressive covariance structures will be used to compare changes in self-determined motivation and self-efficacy between groups. Adherence will be analyzed using *t* tests comparing the mean number of missing step count entries per week and mean number of website log-ins per month between groups. This protocol is registered with ClinicalTrials.gov [NCT02638675].

## Results

Enrollment for the study will be completed in February 2017. Data analysis will commence in September 2017. Study results are to be published in the winter of 2018.

## Discussion

### Overview

Physical activity maintenance is critical for controlling the human and economic burden of chronic disease [1,2]. While incentives have stimulated physical activity behaviors in the past [20-26], only 1 RCT to our knowledge has produced longer term, postintervention improvements [25]. The primary aim of this protocol is to outline the design of an RCT to test whether adding theoretically informed guaranteed incentives to an existing physical activity promotion program can drive physical activity for 12 weeks after guaranteed incentives are removed in a workplace context. Since one of the risks with incentives is that they damage self-determined motivation and thus people’s potential for sustained change [42], the theoretical considerations in this study extend beyond behavioral economics (which merely describes how incentives may be used as a catalyst for change) [19] to include insights from self-determination theory (which describes the conditions under which incentives may produce sustained change) [29]. The literature examining the undermining effect of incentives has mostly considered simple tasks for which initial intrinsic motivation is high, although these findings should not be generalized to lifestyle health behaviors like physical activity where initial intrinsic motivation can be low [43]. In addition, incentive schemes vary greatly in their design and can differentially moderate the undermining effect [43]. While more research is needed, schemes that target less active adults for realistic behavioral outcomes are theorized to support the internalization process and promote quality behavior change [29,30].

According to self-determination theory, incentives may help to build self-determined motivation primarily through their action on self-efficacy, especially for lower active people who exhibit fewer intrinsic motives to begin with (less motivation to crowd out) [44,45]. Regarding physical activity, one hypothesis is that incentives may increase a person’s self-efficacy to become more active by exposing them to a form of physical activity for the first time [29,30,44,45]. Especially if the activity is an achievable one (eg, walk 1000 more steps per day vs walk 10,000 steps per day), individuals may find their confidence to be more active increases after just a few weeks [29,30,44,45]. To align with self-determination theory, then, we decided to (1) target lower active employees only (because they have less/little self-determined motivation to crowd out) [23,24,29] and (2) offer incentives contingent on tailored/realistic step goal achievements (to maximize mastery experiences and increase self-efficacy) [45]. The main theoretical contribution of this protocol therefore is in its application of self-determination theory to the design of the incentive intervention (ie, realistic behavioral targets) and program evaluation (ie, tracking motivation throughout).

The practical implications of this research are also important given the growing popularity of incentive-based wellness programs [42]. First of all, we hope the results of this RCT encourage others to incorporate a simple but important incentive program design nuance by offering rewards contingent on tailored, rather than generic, physical activity goals. The pervasiveness of wearable physical activity monitors in general (eg, smartphones with built-in accelerometers) may make it easier for interventionists or employers to individualize physical activity goals in the context of a health rewards system (by setting goals based on an individual’s own physical activity pattern). Another practical implication of this study may be in encouraging employers to consider guaranteed rewards systems for higher-risk, higher-cost employees only versus the traditional (and largely ineffective) approach of offering low-frequency, low-magnitude chance-based rewards to everyone. To manage budgets, employers often opt for the seemingly more affordable chance-based reward scheme even though there is limited evidence of its effectiveness [20]. Finally, if incentives are not amenable to employees, the intervention will almost certainly fail [46]. Employers deploying incentives should therefore consider any unintended consequences of this novel approach, including (1) perceived unfairness (eg, Why should only lower active employees be rewarded to exercise?), (2) opportunity cost concerns (eg, Should we really be spending money on this?), (3) gaming/cheating (eg, I will cheat by getting my friend to track my activity for me), and (4) low overall acceptability (eg, I don’t want my employer telling me what to do) [47-49]. One way of circumventing the perceived unfairness issue may be in offering minimal chance-based rewards to all employees regardless of physical activity level as well as an enhanced incentive program to employees qualifying as lower active or higher-risk. Supporting such an approach with empirical data may alleviate concerns around unfairness, opportunity cost, and acceptability as well [47-49].

### Limitations

This study protocol is not without limitations. First, given that only employees already enrolled in Change4Life (less than 10% of the eligible employee population) will be assessed for eligibility and invited to participate in the study, the results may not be generalizable. By recruiting only lower active Change4Life enrollees, we will learn more about how a higher risk employee population responds to incentives. Since the hallmark of Web-based health interventions is low engagement [49], there is potential for significant study dropout as well (ceasing daily device synchronization and not participating in assessments), especially among standard care participants. Both intervention and standard care participants will be encouraged to participate in all scheduled assessments (baseline, intervention end point, follow-up) with Can $10 (US $7.40) vouchers. Given the low participant burden and potential for lower active individuals to experience improved health, however, this voluntary incentive-based wellness program may be met with relatively high levels of engagement. Finally, participants will not be blinded to study group allocation, which could contaminate the results. Knowledge of group allocation will be assessed using a study exit survey to monitor this potential confounder.

### Conclusion

The objective of this study is to improve the longer term maintenance of physical activity through a better understanding of how to structure and evaluate incentive programs. Incentives are not a panacea, of course, and may not work for all people, but as part of broader package of interventions and under certain conditions incentives may have a role to play in driving sustained health behavior change.

## Abbreviations

BREQ-3: Behavioral Regulation to Exercize Questionnaire
MVPA: moderate-vigorous physical activity
SEE: Self-Efficacy for Exercise

## References

[1] Janssen I (2012). Health care costs of physical inactivity in Canadian adults. Appl Physiol Nutr Metab.

[2] Li L (2014). The financial burden of physical inactivity. J Sport Health Science.

[3] Krueger H, Turner D, Krueger J, Ready AE (2014). The economic benefits of risk factor reduction in Canada: tobacco smoking, excess weight and physical inactivity. Can J Public Health.

[4] Garber CE, Blissmer B, Deschenes MR, Franklin BA, Lamonte MJ, Lee I, Nieman DC, Swain DP, American College of Sports Medicine (2011). American College of Sports Medicine position stand: quantity and quality of exercise for developing and maintaining cardiorespiratory, musculoskeletal, and neuromotor fitness in apparently healthy adults: guidance for prescribing exercise. Med Sci Sports Exerc.

[5] Conference Board of Canada (2015). Moving Ahead: Workplace Interventions to Reduce Physical Inactivity and Sedentary Behaviour.

[6] The Sanofi Canada Healthcare Survey 2015.

[7] Wang F, McDonald T, Champagne LJ, Edington DW (2004). Relationship of body mass index and physical activity to health care costs among employees. J Occup Environ Med.

[8] van Dongen JM, Proper KI, van Wier MF, van der Beek AJ, Bongers PM, van Mechelen W, van Tulder MW (2011). Systematic review on the financial return of worksite health promotion programmes aimed at improving nutrition and/or increasing physical activity. Obes Rev.

[9] Hutchinson AD, Wilson C (2012). Improving nutrition and physical activity in the workplace: a meta-analysis of intervention studies. Health Promot Int.

[10] Meenan RT, Vogt TM, Williams AE, Stevens VJ, Albright CL, Nigg C (2010). Economic evaluation of a worksite obesity prevention and intervention trial among hotel workers in Hawaii. J Occup Environ Med.

[11] Brown HE, Ryde GC, Gilson ND, Burton NW, Brown WJ (2013). Objectively measured sedentary behavior and physical activity in office employees: relationships with presenteeism. J Occup Environ Med.

[12] Towers Watson (2013). 2013/2014 Staying@Work Report: Canada Summary.

[13] Berry LL, Mirabito AM, Baun WB (2010). What's the hard return on employee wellness programs?. Harv Bus Rev.

[14] Henke RM, Goetzel RZ, McHugh J, Isaac F (2011). Recent experience in health promotion at Johnson & Johnson: lower health spending, strong return on investment. Health Aff (Millwood).

[15] Stock S, Schmidt H, Büscher G, Gerber A, Drabik A, Graf C, Lüngen M, Stollenwerk B (2010). Financial incentives in the German Statutory Health Insurance: new findings, new questions. Health Policy.

[16] Ries N (2012). Financial incentives for weight loss and healthy behaviours. Healthc Policy.

[17] Schmidt H, Stock S, Doran T (2012). Issues in international health policy: moving forward with wellness incentives under the Affordable Care Act. Issue Brief (Commonwealth Fund).

[18] Camerer CF, Loewenstein G, Camerer CF, Loewenstein G, Rabin M (2003). Behavioral economics: past, present, future. Advances in Behavioral Economics.

[19] Loewenstein G, Asch DA, Volpp KG (2013). Behavioral economics holds potential to deliver better results for patients, insurers, and employers. Health Aff (Millwood).

[20] Mitchell MS, Goodman JM, Alter DA, John LK, Oh PI, Pakosh MT, Faulkner GE (2013). Financial incentives for exercise adherence in adults: systematic review and meta-analysis. Am J Prev Med.

[21] Strohacker K, Galarraga O, Williams DM (2014). The impact of incentives on exercise behavior: a systematic review of randomized controlled trials. Ann Behav Med.

[22] Patel MS, Asch DA, Rosin R, Small DS, Bellamy SL, Heuer J, Sproat S, Hyson C, Haff N, Lee SM, Wesby L, Hoffer K, Shuttleworth D, Taylor DH, Hilbert V, Zhu J, Yang L, Wang X, Volpp KG (2016). Framing financial incentives to increase physical activity among overweight and obese adults: a randomized, controlled trial. Ann Intern Med.

[23] Pope L, Harvey J (2015). The impact of incentives on intrinsic and extrinsic motives for fitness-center attendance in college first-year students. Am J Health Promot.

[24] Kullgren J, Troxel AB, Loewenstein G, Asch DA, Norton LA, Wesby L, Tao Y, Zhu J, Volpp KG (2013). Individual- versus group-based financial incentives for weight loss: a randomized, controlled trial. Ann Intern Med.

[25] Charness G, Gneezy U (2009). Incentives to Exercise. Econometrica.

[26] Finkelstein EA, Haaland BA, Bilger M, Sahasranaman A, Sloan RA, Nang EEK, Evenson KR (2016). Effectiveness of activity trackers with and without incentives to increase physical activity (TRIPPA): a randomised controlled trial. Lancet Diabetes Endocrinol.

[27] Burns R, Donovan AS, Ackermann RT, Finch EA, Rothman AJ, Jeffery RW (2012). A theoretically grounded systematic review of material incentives for weight loss: implications for interventions. Ann Behav Med.

[28] Oliver A (2012). A nudge too far? A nudge at all? On paying people to be healthy. Healthc Pap.

[29] Deci EL, Koestner R, Ryan RM (1999). A meta-analytic review of experiments examining the effects of extrinsic rewards on intrinsic motivation. Psychol Bull.

[30] Teixeira PJ, Carraça EV, Markland D, Silva MN, Ryan RM (2012). Exercise, physical activity, and self-determination theory: a systematic review. Int J Behav Nutr Phys Act.

[31] Bassett JD, Wyatt HR, Thompson H, Peters JC, Hill JO (2010). Pedometer-measured physical activity and health behaviors in U.S. adults. Med Sci Sports Exerc.

[32] (2013). Statistics Canada. Canadian Health Measures Survey 2007/2008 to 2012/2013.

[33] Gilson ND, Faulkner G, Murphy MH, Meyer MR, Washington T, Ryde GC, Arbour-Nicitopoulos KP, Dillon KA (2013). Walk@Work: An automated intervention to increase walking in university employees not achieving 10,000 daily steps. Prev Med.

[34] Leahey TM, Subak LL, Fava J, Schembri M, Thomas G, Xu X, Krupel K, Kent K, Boguszewski K, Kumar R, Weinberg B, Wing R (2015). Benefits of adding small financial incentives or optional group meetings to a Web-based statewide obesity initiative. Obesity (Silver Spring).

[35] Saunders TJ, Gray CE, Borghese MM, McFarlane A, Mbonu A, Ferraro ZM, Tremblay MS (2014). Validity of SC-StepRx pedometer-derived moderate and vigorous physical activity during treadmill walking and running in a heterogeneous sample of children and youth. BMC Public Health.

[36] Colley RC, Barnes JD, Leblanc AG, Borghese M, Boyer C, Tremblay MS (2013). Validity of the SC-StepMX pedometer during treadmill walking and running. Appl Physiol Nutr Metab.

[37] Markland D, Tobin V (2004). A modification to the Behavioural Regulation in Exercise Questionnaire to include an assessment of amotivation. J Sport Exerc Psychol.

[38] Wilson P, Rodgers W, Loitz C, Scime G (2006). It?s who I am...Really!? The importance of integrated regulation in exercise contexts. J Appl Biobehav Res.

[39] Resnick B, Jenkins LS (2000). Testing the reliability and validity of the Self-Efficacy for Exercise scale. Nurs Res.

[40] Random.org.

[41] Haff NM, Patel R, Lim R, Zhu J, Troxel AB, Asch DA, Volpp KG (2015). The role of behavioral economic incentive design and demographic characteristics in financial incentive-based approaches to changing health behaviors: a meta-analysis. Am J Health Promot.

[42] Mantzari E, Vogt F, Shemilt I, Wei Y, Higgins JP, Marteau TM (2015). Personal financial incentives for changing habitual health-related behaviors: A systematic review and meta-analysis. Prev Med.

[43] Promberger M, Marteau TM (2013). When do financial incentives reduce intrinsic motivation? Comparing behaviors studied in psychological and economic literatures. Health Psychol.

[44] Sweet S, Tulloch H, Fortier MS, Pipe AL, Reid RD (2011). Patterns of motivation and ongoing exercise activity in cardiac rehabilitation settings: a 24-month exploration from the TEACH Study. Ann Behav Med.

[45] Biddle S, Mutrie N (2008). Psychology of Physical Activity: Determinants, Well-Being, and Interventions.

[46] Volpp KG, Asch DA, Galvin R, Loewenstein G (2011). Redesigning employee health incentives—lessons from behavioral economics. N Engl J Med.

[47] Marteau TM, Mantzari E (2015). The case for pay to quit: A randomized controlled trial of four financial-incentives programmes for smoking cessarion. Nature.

[48] Mitchell M, Goodman JM, Alter DA, Oh PI, Faulkner GE (2014). “Will walk for groceries”: acceptability of financial health incentives among Canadian cardiac rehabilitation patients. Psychol Health.

[49] Mitchell MS, Faulkner GE (2014). On supplementing “foot in the door” incentives for eHealth program engagement. J Med Internet Res.

